# Newspaper Coverage of Snus in an Emerging Norwegian Snus Market 2002–2011: A Content Analysis

**DOI:** 10.1093/ntr/ntab171

**Published:** 2021-08-31

**Authors:** Sæbø Gunnar, Tokle Rikke Iren, Lund Ingeborg

**Affiliations:** Norwegian Institute of Public Health (NIPH), Skøyen, Oslo, Norway

## Abstract

**Background:**

In a context where snus is a legal product, its advertising is prohibited and its prevalence of use has been on the rise among adolescents and young adults, the aim of this article is to identify the extent of snus coverage in Norwegian newspapers and the themes and values communicated about snus therein from 2002 to 2011.

**Aim and methods:**

All major Norwegian newspapers were scanned for articles with “snus” (and relevant connectors) in headings, ingresses, and/or pictures/captions as search criteria. Using the Retriever media monitoring service as a database, the search returned 943 unique articles, which were subjected to quantitative content analysis.

**Results:**

The number of articles per year increases over the period, while their average length decreases slightly. Thematically, the greatest attention is on the extent of “snus use” (occurring in 52.7% of the articles), and then more equally divided between “tobacco policy” (24.5%), “economy/markets” (29.1%), and “health” (28.7%). A total of 48.6% of the articles are “neutral/mixed” in respect of framing, 28.1% are “negative,” and only 20.7% are “positive” in tone. Articles about tobacco policy are more often negative, while articles on economic factors are more often positive. Articles on health are usually negatively focused, or neutral/mixed.

**Conclusion:**

The slight predominance of negative and/or neutral/mixed articles indicates that the newspaper coverage does not glamorize the snus product. However, the sheer amount of (and growth in) articles over time, as well as positive articles available for selective exposure and perception, may nevertheless have contributed to a normalization of snus use.

**Implications:**

Little is known about media coverage of smokeless tobacco and whether editorial mass media glamorize or criticize its use. This study shows that the extent of snus coverage in Norwegian newspapers has increased over time, but also that the framing of Norwegian newspaper coverage of snus has mainly been neutral/mixed or negative toward snus and its use.

## Background

Swedish snus is a form of smokeless tobacco legal in only a few countries, including Sweden, Norway, and the United States. Swedish snus differs from other types of snuff and chewing tobacco by following the GothiaTek standard, which means a significantly lower content of carcinogenic nitrosamines.^1^ Among nicotine products, Swedish snus is at the lower end of the risk scale.^2^ Consequently, snus has a harm-reducing potential compared to cigarettes.^3–6^ Harm-reduction communication is complicated, however, as Swedish snus is manufactured and sold by the Swedish Match tobacco company, and thus subject to the WHO Framework Convention on Tobacco. There is currently considerable controversy surrounding the proposal to accept snus as a harm-reducing measure.

The Norwegian (and Swedish) situation is internationally unique in that snus is not only legal, but its use has increased despite a ban on advertising in 1975 (in Sweden, 1993) and a tobacco display ban in 2010. Simultaneously, smoking prevalence has decreased in both countries. This creates a somewhat different communicative context for tobacco harm reduction compared to most countries (including the United States, which has a low prevalence of smokeless tobacco use, despite advertising being allowed). The prevalence of snus use in Norway doubled from 6.1% in 2002 to 12.6% in 2012, mainly due to an increase among adolescents and young adults, before it flattened out somewhat and then increased again from 2015.^7^ Sweden went through a similar development to Norway, but there snus use among young people started rising as far back as the 1980s. In Finland, there was an increase in the period from 1981 to 2003, despite a total ban on snus sales coming into force in 1995.^8^ The EU banned snus in 1992, but in the UK recent initiatives have argued for a repeal of the snus ban.^9^ Switzerland’s ban on snus was repealed in the summer of 2019. The Norwegian experience illustrates what may happen if snus is allowed to compete with cigarettes in markets where advertising of the product is not permitted.

In a context where snus is legal but product advertising is banned, general media coverage of snus becomes an important channel of information, as social definitions of the pros and cons of use, the product’s health risks, and how it should be regulated, will largely depend on mass-mediated communication.^10^ This applies both to the governors and the governed. Stakeholders in tobacco control take to the news media to gain currency for their initiatives and objectives, and to achieve credibility and legitimacy for their proposals.^11,12^ Similarly, the snus industry may recognize the value of getting their products featured in the media, as they are at the mercy of means other than traditional advertising for creating a buzz about their products.

However, mass media is not just a neutral channel of information, but also a communication facility with independent powers.^13^ The agenda-setting function of the media refers to its power to decide what the public is talking about at any given time and, specifically, how it introduces tobacco issues to the public.^14^ Editors and journalists influence tobacco-opinion formation in society through their selection of tobacco issues^15^ and through the process of framing the media content on tobacco.^16^ The audience learns not only about a given topic, but also about the importance of the theme, based on content size, positioning, and wording. The journalistic norm is neutrality through balanced presentation, and editors often use sources with divergent views and agendas to achieve this balance.^17^ Different actors convey different messages, and some have a greater impact than others.^18^

Although all types of media occasionally publish information useful to citizens about smokeless tobacco and its risks, the newspaper is probably the medium where snus is discussed in the most systematic and elaborate way and with broad coverage.^19,20^ In the current media landscape, newspapers also represent an important link between the traditional, unidirectional mass media and contemporary interactive social media, as they become increasingly integrated into platforms such as Facebook and Twitter. It is therefore important to explore whether there are changes in themes, values, and actor constellations in newspaper coverage of snus over time.

### Previous Studies

A study of tobacco images and texts in Norwegian magazines and newspapers found that most editors had no procedures or restrictions in respect of publishing indirect tobacco advertising or images of people smoking.^21^ Stories promoting smoking were far more common than stories concerning health hazards (71% versus 29%). We do not know if similar patterns apply to snus coverage.

Internationally, several content analyses of newspaper writings on tobacco have been conducted, but only occasionally have articles about snus been studied. When snus has been a topic, it has usually been studied as part of the broader concept of “smokeless tobacco.” ^22,23^ In a study of US news coverage of smokeless tobacco in the period 2006–2010, the researchers set out to identify issues related to snuff, risk references, and perspectives/bias (“slants”) in the major national and regional newspapers and news wires.^24^ The study showed that the majority of stories were negative and critical towards smokeless tobacco.

### Research Problem

The aim of the present study is to describe the coverage of snus in Norwegian newspapers in a 10-year period characterized by a doubling in snus use, and changes in the demographic composition of users, most notably due to more snus use by adolescents and young adults.^25,26^ Specifically, we will assess the magnitude of coverage and identify themes and values communicated about snus, study the diversity of messages, and identify the actors representing various topics and the factual knowledge base for the coverage. The study will provide an empirical basis for investigating the role of newspapers in glamorizing and normalizing snus use, and for assessing the extent to which messages about snus in this time period signaled positive or negative framing of health values.

To explore these issues, we have conducted a descriptive content analysis of the newspapers’ overall message system, to reveal structures and changing patterns in the newspaper narratives over time.

## Methods

### Data

The study was based on the coverage of the snus phenomenon in Norwegian newspapers from 01 January 2002 to 31 December 2011. Since our aim was to identify the overall message system of snus that the Norwegian population had been exposed to via newspapers during this period, we strived for as complete a universe of articles as possible. The data was collected by searching the Retriever media monitoring service, which is essentially complete for all major newspapers (circulation of over 10 000 copies). The following search string was employed (in Norwegian): “snus * AND (smoke * OR cigarette * OR tobacco * OR roll-your-own * OR Health Directorate * OR SIRUS *)” (SIRUS was the abbreviation for the Norwegian Institute for Alcohol and Drug Research). This search combination produced a higher number of hits than when snus was combined with each feature separately.

The inclusion criterion was that the word “snus,” in the relevant meaning of the word, was included in the headline or the ingress or that an image of snus appeared as an illustration. In other words, we only included articles where snus was the *main topic*. This limitation is justified by capacity considerations in respect of coding work and by the journalistic storytelling norm of presenting the main point of a story first (the norm of “the inverted pyramid”). This may also be justified based on reception considerations. The common way to read an article is from the start (or from the “top”), and many readers read no more than the headlines and captions.^27^

Based on these criteria, the search resulted in 943 unique hits in Norwegian newspapers, which included national press, regional newspapers, and local newspapers, on both paper and web platforms.

Based on the methodological technique for quantitative content analysis,^28^ all articles were reviewed and coded in SPSS by the second author, using article as analytical unit. All articles included were printed and archived, to ensure verifiability. To check coding for reliability, the first author conducted a reliability test of 10% of the articles (randomly drawn). Cronbach’s alpha varied between .83 and 1.00 for the variables applied in the present analysis, which must be considered satisfactory.

As all newspaper articles are publicly available, no ethical permissions were required.

### Measures

#### Newspaper Variables

Information about extent of coverage and journalistic priorities was registered from article characteristics. In addition to newspaper name and date of article, we coded “type of article” (news vs other journalistic genres), whether the article was “original or reproduced from news agencies/wires” and “article length” (number of words).

#### Snus Variables

To address topical issues and stakeholder interests, a set of exploratory snus variables was established, based on issues prominent in the public debate. The *main themes* fell into four categories: “snus use” deals with issues related to use and distribution of snus in adults and youth, user rituals, and use in leisure and in school. “Tobacco policy” focuses on policy suggestions and initiatives regarding restrictions and measures to reduce snus use and make users aware of health risks and harm reduction. “Economy and market” were articles relating to market conditions, cross-border trade, sales trends, and investments. Finally, “health” concerned articles focusing on the health hazards of snus use either absolute or relative to other tobacco use, primarily smoking.

#### Framing

Framing refers to the overall key message of the article. Cases coded with “positive” frames were those that portrayed snus as pleasurable, harmless, or superior to cigarettes. Some cases also described celebrities who used snus, or included statements involving criticism of limits and restrictions on snus use or snus sales. Articles coded as “negative” were those that portrayed snus use as hazardous or unattractive. Typical examples included reports of harm to health, restrictions or prohibitions, critique of the tobacco industry, and opinions advocating increased tobacco control. Cases with a “neutral/mixed” frame included descriptive cases or news accounts, short paragraphs with no clear key message, as well as longer reports from multiple angles. Several cases included both positive and negative statements, for instance, arguments pro and contra and/or stakeholders with conflicting interests represented in the same article, which gave the overall impression of the article carrying a balanced view. Cases that could not be classified into the above categories were coded as “unclassifiable.”

When it came to who is represented in the articles, we identified the *actor voice* in each article. This refers to the sender position created in the text; it may be the interviewee or the commentator. The following twelve categories were utilized: “industry representative,” “celebrity,” “sports/athlete,” “researcher,” “politician,” “bureaucrat,” “NGO representative,” “physician,” “school pupil/student,” “teacher/headmaster,” “journalist” and “unspecified citizen.”

Finally, we recorded the *knowledge base* (factual basis) for each article. This variable was divided into three categories: “research/documentation,” meaning articles that referred to, or were based on, research reports or other documentation, “initiatives/input,” which could be, for example, politicians or interest groups, and “remarks/opinion,” i.e. letters to the editor from citizens, statements of schoolchildren and the like.^29^

To consider multidimensionality, all categories of the snus variables were coded as multiple choices based on a logic of “occurring/not occurring,” with the exception of the “framing” variable, where we used mutually exclusive categories to enforce identification of one overall key message.

### Statistical Analysis

Descriptive statistics (univariate frequency tables and bivariate crosstabs) were applied. As categories are overlapping, the reported percentages do not necessarily add up to 100%. To visualize stability and change over time we included trend lines. The linear trend line is a regression line based on the least-squares method. As the data includes nearly the total universe of articles, inferential statistics, i.e. significance testing, were not applied.

The analyses were performed using SPSS 25 and Excel.

## Results

### Journalistic Priorities and Extent of Coverage

The number of articles about snus increased during the period 2002–2011, with the most conspicuous expansion (a doubling of articles) happening from 2002–2003 to 2004–2005 (Table 1). 64.6% of the articles were original publications, while 63.0% were news reports. In terms of trends, there was a slight decrease over time in the average size of articles and a somewhat lower tendency for articles to be original publications, while the tendency to belong to the news genre was stable (trend lines shown in Supplementary Materials).

**Table 1.** Journalistic Priorities by Year

	2002–03	2004–05	2006–07	2008–09	2010–11	Total
Original or reproduced article						
% originally produced	72.4	71.1	55.4	62.1	65.3	*64.6*
% reproduced from agencies/wires	27.6	28.9	44.6	37.9	34.7	*35.4*
Type of article						
% in the news genre	64.3	62.4	58.2	68.1	61.5	*63.0*
% in other genres	35.7	37.6	41.8	31.9	38.5	*37.0*
Size of articles (number of words)						
Means	469	441	399	383	448	*423*
Median	383	403	351	320	359	*321*
(*N*)	(98)	(197)	(177)	(232)	(239)	*(943)*
Percent of total	10.4	20.9	18.8	24.6	25.3	*100.0*

### Frequencies of Themes, Frames, and Knowledge Bases

**Figure 1.**
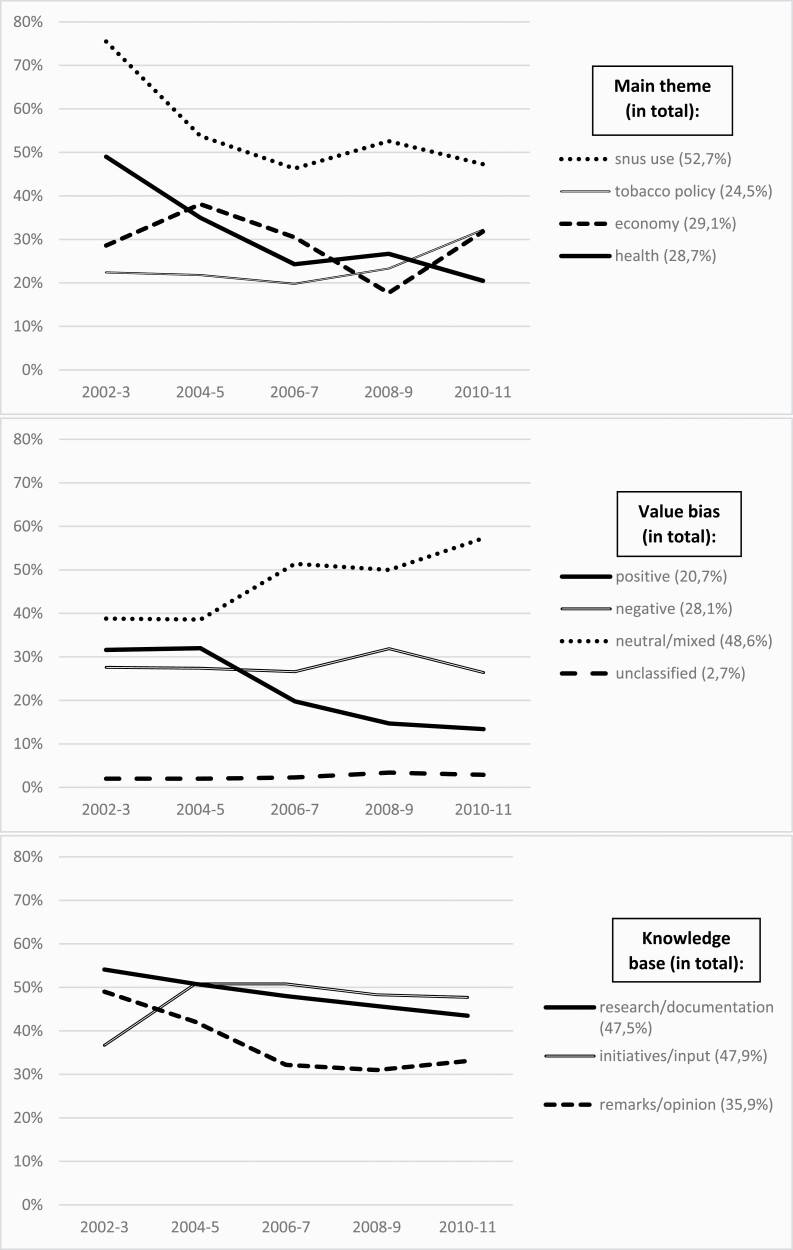
Main theme, value bias, and knowledge base over time. Percentages.

Thematically, the largest group of newspaper articles fell within the thematic category “snus use” (52.7% in total), while there was a more equal split between the categories “tobacco policy” (24.5%), “economy/markets” (29.1%) and “health” (28.7%) (Figure 1). However, while “snus use” and “health” gradually have become less important themes over time, “tobacco policy” has become more important in recent years, while the theme “economy/market” has fluctuated.

Most articles (48.6%) were neutral/mixed in respect of frames, and only 20.7% were positive (Figure 1). Neutral/mixed articles became more prevalent over time, while positive articles became less prevalent.

Almost half of the articles carried a reference to “research/documentation” as a factual basis (Figure 1). This tendency slightly decreased over the studied time-period. References to “initiatives/input” were also widespread and tended to increase slightly from 2002–2003 to 2010–2011. The least commonly used factual basis was “remarks/opinion.”

### Relationships Between Themes, Frames, and Social Actors

**Figure 2.**
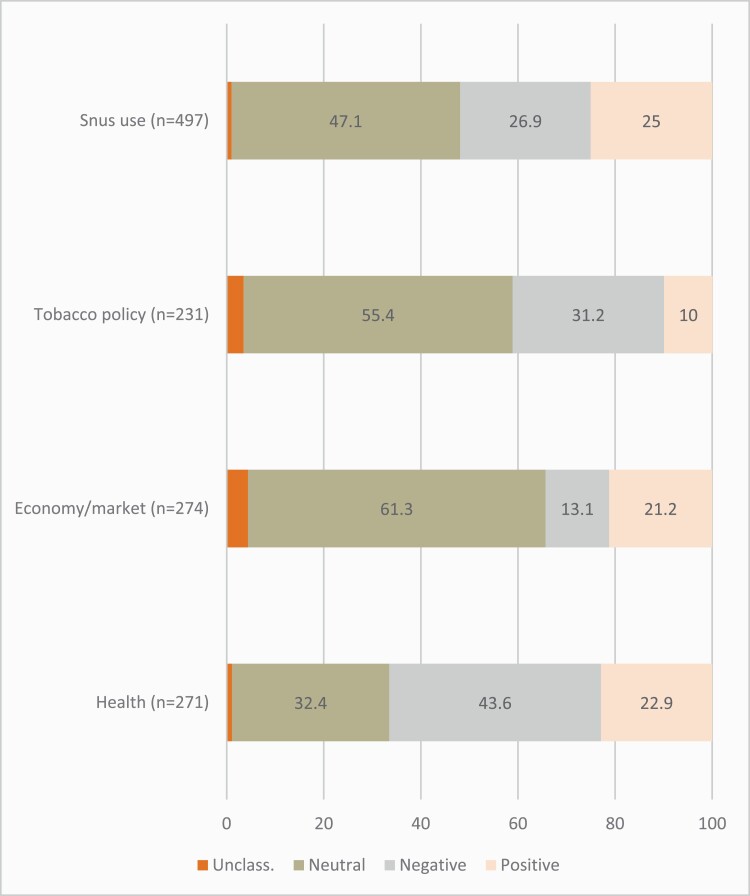
Value bias by snus themes. Percentages.

Articles on snus use had no apparent value bias, with 47.1% of them being neutral/mixed and the rest equally split between positive (25.0%) and negative (26.9%) framing (Figure 2). Stories about snus policies tended to be negative (31.2%, vs. 10% positive articles), while articles relating to economic matters more often conveyed positive connotations (21.2%). The largest proportion of negative focus (43.6%), and the lowest proportion of neutral/mixed focus (32.4%), were found in articles on health aspects.

The most frequently occurring actor voices in the articles were researchers (30%), journalists (25%), snus industry/line of business (22%), bureaucrats (21%), school pupils (15%), citizens (14%), NGOs (14%), and politicians (11%) (Table 2).

**Table 2.** Occurrence of Social Actors, by Theme and Value Bias^a^ of the Article. Percentages (Multiple Choices of *N*)^b^

	Snus use			Tobacco policy			Economy/market			Health			Total
	Pos.	Neut.	Neg.	Pos.	Neut.	Neg.	Pos.	Neut.	Neg.	Pos.	Neut.	Neg.	
Industry	28	16	3	44	32	4	83	52	17	24	19	6	*22*
Celebrity	10	3	2	-	-	1	7	-	-	5	4	3	*3*
Sports/athlete	8	7	9	1	1	3	2	1	-	6	6	3	*5*
Researcher	32	35	14	17	15	6	16	22	17	59	57	41	*30*
Politician	4	12	8	17	34	31	3	14	25	10	5	6	*11*
Bureaucrat	7	19	24	9	33	29	10	29	17	13	23	18	*21*
NGOs	5	11	21	26	12	31	7	8	44	10	17	26	*14*
Physician	5	10	10	-	4	6	3	5	8	11	27	22	*10*
School pupil	25	25	25	13	13	14	—	5	8	2	9	8	*15*
Teacher/headmaster	3	17	16	-	12	14	—	1	8	-	1	1	*8*
Journalist	37	27	16	35	32	19	33	32	22	29	21	16	*25*
Citizen	26	14	15	30	13	8	28	11	6	16	17	7	*14*
N=	(125)	(235)	(132)	(23)	(128)	(72)	(58)	(168)	(36)	(63)	(86)	(119)	*(918)*

^a^Articles with unclassifiable value bias excluded (*N* = 25).

^b^Decimals omitted to increase readability.

The snus industry was primarily represented in stories about economy/markets and policies, politicians and bureaucrats in articles on policies, while researchers usually addressed health conditions (Table 2). Furthermore, the snus industry was more commonly represented in articles with positive frames and less commonly represented in articles with negative frames. The converse was true for representatives from various NGO organizations in tobacco control, who mostly occurred in negatively framed articles and seldom in articles with a positive angle. The journalistic voice was more commonly represented in articles with a positive framing.

## Discussion

### Main Findings

In this study, we have identified the dominant messages about snus, as expressed in Norwegian newspapers’ coverage of snus-related matters over a 10-year period (2002–2011). The most common theme was “snus use,” while “tobacco policy” was the theme that increased the most in this decade. The majority of articles were neutral/mixed or negative in respect of frames, indicating relatively few unambiguously glamorous messages about snus. Furthermore, there was a tendency for a decrease in articles that were positive about snus during the study years.

### Framing of the Snus Phenomenon

In the period of study, there was a strong growth in snus use in Norway, especially among young people and among young men in particular.^7^ Given the advertising ban, the tobacco display ban (from 2010), the 18-year minimum age, and a consistently negative framing of snus by health authorities, the strong growth in snus use from 2002 to 2011 might partly have been fuelled by positive symbolic content in the media. As demonstrated by earlier findings, the media, in all its forms, is likely to shape individuals’ perceptions, attitudes, and behaviors towards tobacco.^10,30^ However, given the predominance of neutral/mixed or negative content found in articles, this study does not indicate any glorifying of snus by Norwegian newspapers. These results are in contrast to an earlier study, which concluded that the coverage of smoking in the Norwegian press was predominantly positive,^21^ but in accordance with a similar study from the United States.^24^

The main topics are raised in repetitive stories dispersed over time and successively supplied with small new elements in the form of research findings, market developments, product developments, user trends etc.^31^ The newspapers do not convey one simple and uniform thematic vision of snus, but rather multiple, and relatively independent, views, which are quite broadly (and repeatedly) represented over time. In sum, the overall message system tends to be heterogeneous rather than homogenous.

The most interesting tension, both analytically and in terms of framing, is linked to the stories about health. These stories are polarized and largely “incoherent,” and involve both more uniformly negative *and* more unambiguously positive news media attention than applies to the other themes. While various, and sometimes conflicting, angles tend to be common in all newspaper media,^16^ we particularly observed this in health-related topics. For example, these two headings were found in the same newspaper from one year to the next: “Snus is good for public health” (*Adresseavisen* 09/01/2007) and “Snus can cause cancer” (*Adresseavisen* 08/09/2008). Another example: “Yes, snus is hazardous” (*Aftenposten* 04/12/2008), while five months later this headline appeared “Thinks that snus can save lives” (*Aftenposten* 04/21/2009).^31^ Such to-the-point wording, bold typefaces, and tabloid headlines are designed to attract readers. The same newspaper can thus communicate that snus is “good” for public health, and that snus is carcinogenic and hazardous to health. All the wordings may individually be correct, but the overall message still appears equivocal.

We find clear associations between topics, framing, and actor voices in the stories. The patterns uncovered are relatively predictable, in that they are clearly sector-based: the snus industry is the main source represented in positive stories about “economy/markets,” politicians and NGOs in the negative articles on “tobacco policy” and researchers in the articles on “health,” irrespective of framing.

Interestingly, the journalistic voice is commonly represented in the positive articles, indicating that some journalists in commentaries tend to side with those promoting snus. Elsewhere, we find that newspapers overall seem to strive for objectivity, with various viewpoints often promoted via actors presenting different messages in the same article. About half of the articles were thus deemed neutral, in the sense of balanced—although they contained both positive and negative representations. In an era where the media often are accused of bias in news reporting, it is also noteworthy that half of the articles were based on research and documentation.

### The Significance of Increased Coverage: Normalization of Snus and Snus Use?

The analysis has shown an increase in the extent of coverage of snus in the 10-year period we are studying. Although this increase corresponds with increases in use and sales, this does not imply that the Norwegian newspapers’ writings about snus have been a direct driver of these increases. We cannot draw conclusions from our analysis about causality or directions of influence—that is, whether more newspaper coverage leads to more use, or whether more use leads to more newspaper coverage. What we have shown is a correspondence between increased use and increased news media coverage.

In this context, can news media still have contributed to a normalization of snus use? Newspapers exert considerable social power by selecting which matters are placed on the public and political agenda, which also contributes to playing various actor groups against each other.^32^ As we have seen, stories about snus are characterized by various slants, often conflicting or negative. Despite these messages not always being overtly positive, such newspaper articles may nevertheless have a promotional effect, since interviewees “share” their experiences, which in turn may contribute to normalization of snus use. Through the agenda-setting attention that *any* coverage provides for the snus product, even “neutral/mixed” and “negative” articles may contribute to create a buzz and interest in snus. Media influence is not entirely dependent on the message carrying an unambiguously positive connotation (as powerfully expressed in the phrase “all PR is good PR”).

The concept of normalization refers to an action moving from being deviant, or reserved to some subcultural communities, to becoming “mainstream” and common.^33^ In normalization processes, dissemination of the phenomenon in question is thus vital. Although snus is a legal product with a long history in the Norwegian market, there are now more users of snus compared to the 1990s. The demographic characteristics of people using snus have also changed, as the proportion of young snus users increased considerably during the investigation period.^7^ Many of these snus users differ from smokers by holding higher social status and having more active lifestyles.^25,26^ Simultaneously, there are fewer and fewer “denormalized” smokers (see Supplementary Materials). These developments are also reflected in the newspaper narratives. It is particularly with regard to making the snus product *visible* in society (and thereby legitimizing both the product and its use) that newspaper media may enact a “normalizing” effect.

The constant telling (and retelling) of increasing snus sales and use as a trend is another factor that indicates normalization and justification of snus use. The coverage of increased use may also have contributed to the perceived norms concerning snus use having changed, perhaps making snus use appear more common to more people today than in the 1990s. While we cannot conclude that newspaper coverage of snus has created a “bandwagon effect” based on our newspaper content data only, a hypothesis for future research is that the coverage may have added to a perception that more people use snus than is actually the case, which in turn may have resulted in even more new snus users.^34^ (One more issue that merits future research attention is the possible influence of the snus industry on newspaper coverage).

### Increasing Snus Use and Tobacco Policies

While increasing normalization of snus is considered “epidemic” and highly problematic from a health policy point of view that emphasizes total freedom from tobacco on the precautionary principle, it is less problematic from an alternative “harm reduction” point of view.^35^ There is currently consensus in the scientific community that the health hazards of snus use are considerably fewer and lower than those of cigarette smoking. Influential collective actors such as the FDA^36^ and the American Cancer Society^37^ in the United States and the Royal College of Physicians^4^ in the United Kingdom now all embrace the idea of tobacco harm reduction. From a public health perspective, getting established smokers to switch to snus will most likely give a positive “net effect.” ^38^ A combination of increasing snus use with decreasing smoking is also likely to benefit public health, as illustrated by the Swedish experience.^39^

### Limitations and Strengths of the Study

There are some limitations to this study. As concerns the database, Retriever is not an entirely complete database of all Norwegian newspapers. Some small newspapers are not included or are only included for some of the periods under study. However, there is little reason to believe that the stories we may have missed from these minor newspapers differ systematically in themes and perspectives from the data we do have. The data set we have at our disposal is almost complete for all major newspapers (circulation of over 10 000 copies).

Concerning sampling, we cannot ignore that some articles, even with such an extensive search string as described in the methods section, may still not have been encompassed by our search criteria. A strength of this research design, however, is that it provides a valid representation of the composition of the message system of snus over time.

## Conclusion

The number of snus articles per year in Norwegian newspapers increases from 2002 to 2011, while their average size decreases slightly. Variants of the same themes about snus recur throughout the study period, and newspapers convey contradictory messages and values about the risks to health of snus. The slight predominance of negative and/or neutral/mixed articles indicates that the newspaper coverage does not glamorize snus. Still, it cannot be ruled out that the sheer amount of (and growth in) articles over time, as well as positive articles and angles available for selective exposure and perception, may nevertheless have contributed to normalization of snus use in Norway.

## Supplementary Material

A Contributorship Form detailing each author’s specific involvement with this content, as well as any supplementary data, are available online at https://academic.oup.com/ntr.

ntab171_suppl_Supplementary_Materials

ntab171_suppl_Supplementary_Taxonomy_Form
