# Supplementary material for: Newspaper Coverage of Snus in an Emerging Norwegian Snus Market 2002–2011: A Content Analysis
Source: Nicotine Tob Res. 2021 Aug 31;24(2):212–9. doi: 10.1093/ntr/ntab171 (PMC8807211; doi:10.1093/ntr/ntab171)
Supplement: ntab171_suppl_Supplementary_Materials [file ntab171_suppl_supplementary_materials.docx]

**Supplementary material: Journalistic priorities over time. Numbers from table 1 visualized as linear trend lines.**

**Figure. Smoking and snus use (daily or occasional) in Norway 1999-2019. Prevalence, 16-74 years. Source: Statistics Norway/NIPH.**

**
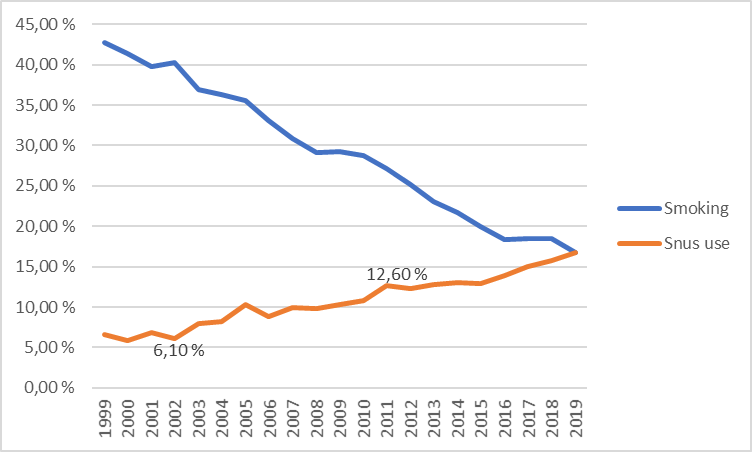
**
